# A Rapid Prediction Method of Moisture Content for Green Tea Fixation Based on WOA-Elman

**DOI:** 10.3390/foods11182928

**Published:** 2022-09-19

**Authors:** Tianmeng Lan, Shuai Shen, Haibo Yuan, Yongwen Jiang, Huarong Tong, Yang Ye

**Affiliations:** 1 College of Food Science, Southwest University, Chongqing 400715, China; 2 Tea Research Institute Chinese Academy of Agricultural Sciences, National Engineering Research Center for Tea Processing, Hangzhou 310008, China

**Keywords:** miniaturized near-infrared spectroscopy, green tea fixation, moisture content, whale optimization algorithm, Elman neural network

## Abstract

Fixation is the most critical step in the green tea process. Hence, this study developed a rapid and accurate moisture content detection for the green tea fixation process based on near-infrared spectroscopy and computer vision. Specifically, we created a quantitative moisture content prediction model appropriate for the processing of green tea fixation. First, we collected spectrum and image information of green tea fixation leaves, utilizing near-infrared spectroscopy and computer vision. Then, we applied the partial least squares regression (PLSR), support vector regression (SVR), Elman neural network (ENN), and Elman neural network based on whale optimization algorithm (WOA-ENN) methods to build the prediction models for single data (data from a single sensor) and mid-level data fusion, respectively. The results revealed that the mid-level data fusion strategy combined with the WOA-ENN model attained the best effect. Namely, the prediction set correlation coefficient (Rp) was 0.9984, the root mean square error of prediction (RMSEP) was 0.0090, and the relative percent deviation (RPD) was 17.9294, highlighting the model’s excellent predictive performance. Thus, this study identified the feasibility of predicting the moisture content in the process of green tea fixation by miniaturized near-infrared spectroscopy. Moreover, in establishing the model, the whale optimization algorithm was used to overcome the defect whereby the Elman neural network falls into the local optimum. In general, this study provides technical support for rapid and accurate moisture content detection in green tea fixation.

## 1. Introduction

Green tea is one of the six major types of tea in China, loved by the Chinese for its fresh taste, refreshing aroma, and high level of polyphenols, which have been proven to benefit health [1]. Fixation is the most critical step in the green tea process [2], during which the green tea is heated to remove part of the internal moisture, emanate green gas, form green tea flavor, and deactivate the enzyme activity to prevent browning reactions [3]. Moisture content directly affects the physical state and biochemical reactions of fixation leaves and plays an important role in the formation of tea quality [4]. The high moisture content of fixation leaves corresponds to a grassy taste of tea infusion, or its low moisture content corresponds to a pasty taste of tea infusion. The tea infusion has a fresh taste only when the moisture content is suitable [5]. Therefore, moisture is usually used as an essential indicator for evaluating the fixation degree, mainly based on human experience, to determine whether green tea fixation is appropriate [6]. However, this is prone to human judgment discrepancies affecting the quality of green tea, and it cannot meet the large volume of production. Hence, rapid and nondestructive moisture detection during the green tea fixation process is helpful to evaluate the fixation degree and ensure a standardized production objectively.

Currently, the literature presents limited research on the rapid and nondestructive detection of the moisture content of fixation leaves with near-infrared spectroscopy (NIRS), a typical nondestructive detection technique. Indeed, NIRS easily obtains information on hydrogen-containing groups by scanning the NIR spectrum of a sample to detect its structure and composition [7]. NIR has been used extensively in tea research, e.g., tea quality identification [8,9], key components detection in tea [10,11,12], and online testing during tea processing [13,14]. However, these studies employ laboratory-grade benchtop spectrometers, which are expensive, nonportable, and complex to operate, and, therefore, cannot be used directly in production. Thus, inexpensive, portable, and simplified equipment is more practical. Recently, a miniature mobile NIR spectroscopy system based on a smartphone has attracted significant research attention [15,16] due to its low cost, small form factor, smartphone connectivity, applicability in various complex applications, and broad scope for development, which poses an application prospect based on these advantages.

Therefore, for the first time, this study utilizes this miniature device to collect near-infrared spectra of fixation leaves during green tea processing, to verify the device’s applicability in the green tea fixation process.

Computer vision technology (CV) is another commonly used nondestructive detection technology that has been used for research related to tea in many ways, including picking [17,18] and tea processing detection [19,20]. It has been shown that fusing image information with spectral information can improve the model’s prediction accuracy compared to single-modality information [8,19,21]. This is because NIRS can characterize the sample’s internal chemical composition but not its external image; thus, computer vision techniques can compensate for this. This demonstrates that multimodal data fusion is gradually becoming a new direction in tea-related research. Still, the corresponding studies on multimodal fusion techniques in tea detection and analysis are limited. Furthermore, there are even fewer studies based on multimodal fusion to detect the moisture content in green tea fixation.

Neural networks are widely recognized for their excellent predictive ability and are widely used to build nonlinear models [22]. Hu et al. proposed an improved deep convolutional neural network (CNN) for the tea leaves disease identification method, which had a higher average recognition accuracy than traditional machine learning methods [23]. McKenzie et al. characterized green, white, black, Oolong, and Pu-erh teas by the total mineral content of tea leaves and demonstrated that the classification performance of probabilistic neural networks (PNNs) was superior to the linear discriminant analysis (LDA) [24]. Shen et al. used an Elman neural network to rapidly predict the moisture of wilted leaves of black tea with a prediction correlation coefficient of 0.99314 and a residual prediction deviation of 11.8108 [25]. The Elman neural network adds the context layer to the hidden layer based on the basic structure of the BP neural network, affording the whole network to adapt to the time-varying characteristics and presenting a stronger computational ability [26]. However, the training process for optimizing weights and thresholds is stochastic and can lead to unstable predictions [27,28]. Nevertheless, the whale optimization algorithm has a good global optimization-seeking capability [29] and can optimize the training process for the weights and thresholds of the Elman neural network.

In previous studies, aroma and color were used as indicators to online evaluate green tea fixation degree [6,30]. However, in this study, we use moisture as the online monitoring object of the fixation and first combine machine vision and miniature near-infrared spectroscopy to quantitatively predict moisture. We also compare the advantages of the Elman neural network based on whale optimization algorithm with current mainstream models for obtaining a model with higher accuracy and robustness, which provides a new idea for intelligent large-volume production of green tea.

## 2. Material and Methods

### 2.1. Sample Preparation

**Figure 1:**
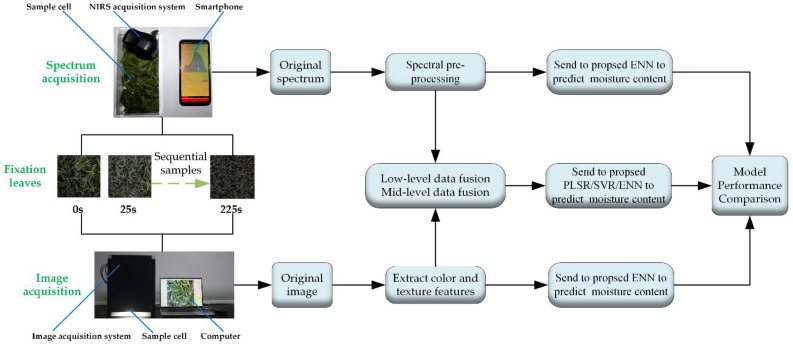
Flow chart of this study.

The fixation samples were prepared at the Shengzhou Comprehensive Experimental Base, Shaoxing, Zhejiang Province, on 23 March 2021. The variety of the tea leaves was Zhongcha 108, and the tenderness was one bud and one leaf. The fresh leaves were spread on a withering rack with a leaf thickness of 2–3 cm, at 19 °C room temperature and 65% humidity. After spreading for 12 h, the fixation began. Fixation was carried out in a roller fixation machine with a rotation speed of 36 r/min. Every 25 s, enough samples were taken from the roller to collect data, ten times in total. The experimental procedure is illustrated in Figure 1.

### 2.2. Data Acquisition

#### 2.2.1. Spectral Data Collection

**Figure 2:**
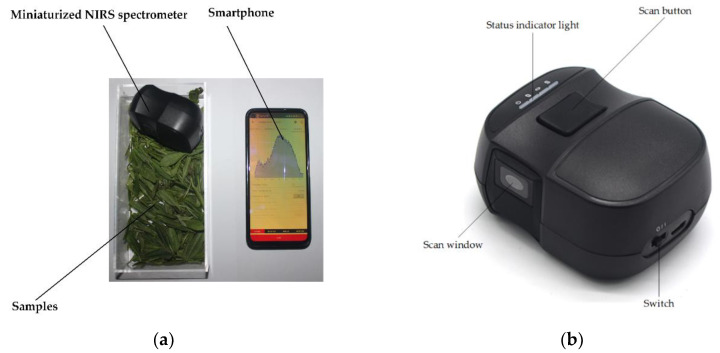
Miniaturized NIRS system, (**a**) spectrum acquisition process, and (**b**) miniaturized NIRS spectrometer [25]. For spectrum acquisition, the scan window of the miniaturized NIRS spectrometer is placed close to the samples and the scan button is then pressed, and the acquired spectrum is displayed on the smartphone.

This study collected spectra using a portable miniature NIR spectroscopy system consisting of a Huawei Honor 30 s smartphone (Huawei Technologies Co., Ltd., Shenzhen, China) and a miniature NIR spectrometer (NIR-S-R2; Innospectrum Corporation, Taiwan, China) with a spectral range of 900–1700 nm. The spectrometer has an external dimension of 75 mm × 58 mm × 26.5 mm, weighs about 77 g, and has a 10–12 nm resolution. The wavelength accuracy of the spectrometer is 1–2 nm, and the signal-to-noise ratio is 6000:1. The ISC NIRScan smartphone app was supplied by the manufacturer (InnoSpectra Corporation, Taiwan, China), and was used solely for acquiring and storing spectral data in CVS format. Subsequently, the collected data were transferred from the smartphone to the computer in CVS format and converted to matrix format in Microsoft Excel (Microsoft Corporation, Washington, DA, USA). Finally, the processed data were used to build a quantitative prediction model in Matlab (MathWorks Inc., Natick, MA, USA). The system setup is depicted in Figure 2.

The miniaturized NIR spectrometer is switched on and prewarmed for 30 min before starting the acquisition to ensure system stability. The data acquisition process started with a whiteboard calibration, followed by three randomly scanned points from the surface of the fixation leaves. The average of the data from three scans was considered as one spectrum of that sample. Thirty-four spectra of the fixation leaves under each moisture were taken. This study obtained 340 spectra of fixation leaves at different fixation timings.

#### 2.2.2. Image Acquisition

**Figure 3:**
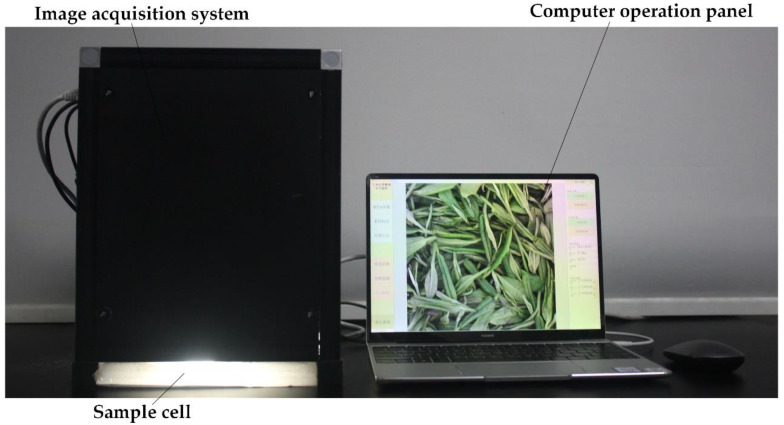
Image acquisition process. For image acquisition, the sample is placed in the sample cell with an industrial camera at the top and the image is acquired on a computer operating system.

Our computer vision system included an image sensor, professional industrial camera, dark box, arc light source, computer, and GUI software processing system. The camera lens was fixed to the top of the image acquisition system to ensure a constant angle and distance. The image acquisition system was turned on for 30 min to warm up before starting the acquisition to ensure system stability. The samples were laid out on A4 white paper and placed in the sample cell with a professional industrial camera (FI-S200C-G; 4 mm low distortion lens) at the top, which covered the camera’s field of view. The shutter was controlled by software on the computer to take images. The system setup is depicted in Figure 3. Thirty-four pictures of the fixation leaves under each moisture were taken, giving a total of 340 images for subsequent data processing. The sensor was a 1/2.8 CMOS image sensor, the resolution was 1080 × 1080 pixels, and the exposure time was set to 0.18 ms. The light source adopted the DOME monochrome pure white arc light source setup, and the average light intensity at the bottom was about 1000 lux (lx). The images were stored in BMP format.

#### 2.2.3. Determination of Moisture Content of Fixation Leaves

After collecting the spectral and image data, the moisture content of the fixation leaves at different fixation times was measured using a rapid moisture analyzer. Each time a 5 g sample was weighed and measured, the measurement was repeated three times, and the average value was taken as the moisture content of the fixation leaves. The moisture content of the samples ranged from 15.97% (225 s of fixation time) to 68.17% (0 s of fixation time).

### 2.3. Data Processing

#### 2.3.1. Spectral Preprocessing

We chose the data in the 980–1670 nm range for further analysis, as the noise interference was beyond the starting and ending points of the considered wavelength range. To eliminate the effects of astigmatism and noise during spectrum acquisition, the raw spectra were preprocessed with multiplicative scatter correction (MSC), max–min normalization (max–min), and standard normal variate (SNV). The preprocessed spectral data were used to build an Elman neural network prediction model and were compared against the Elman neural network model built from the original spectral data to determine the optimal spectral preprocessing method.

#### 2.3.2. Image Feature Extraction

In this study, a 1080 × 1080 pixel area was automatically segmented from the images using a laboratory CV system for the images of fixation leaves. Subsequently, the image feature averages (color and texture) of this region of interest were extracted using the image processing toolbox in Matlab R2021b (The Mathworks, Inc., Natick, MA, USA), and a total of 17 image feature variables were obtained.

The color characteristics included nine variables: R (red), G (green), B (blue), H (angle hue), S (saturation), V (value), L* (L* indicates diffuse white or lightness), a* (a* indicates the position between red and green), and b* (b* from yellow and green). A total of eight texture feature variables were extracted based on the gray-level co-occurrence matrix analysis, which was average gray level (m), uniformity (U), entropy (e), correlation (c), inertia (i), variance (v), inverse difference moment (IDM), and energy (en).

#### 2.3.3. Data Dimensionality Reduction and Data Fusion

Principal component analysis (PCA) is a commonly used feature extraction method that converts a set of potentially correlated variables into a set of linearly uncorrelated variables, where the converted variables are called principal components (PCs). PCA aims to extract the primary orthogonal PCs, which are considered to replace the original data when the accumulated contribution of the selected principal components reaches a specific value. PCA can reduce the data dimensionality and the model’s runtime and remove the interference of irrelevant variables to make the model prediction results more accurate [31]. In this study, the accumulated contribution of SNV preprocessed spectra in the first five PCs reached 99.41%, and therefore we consider that it could represent the overall SNV preprocessed data. Similarly, the accumulated contribution of the image extracted data in the first two PCs reached 99.96%, which could replace the overall image data.

Data fusion involves fusing data with different properties, which can improve the data’s information quality. Data fusion is divided into low level, middle level, and high level. High-level data fusion is mostly applied to classification problems [32], whereas this work focuses on quantitative prediction. Hence, this study used the low-level data fusion strategy (LDF) and middle-level data fusion strategy (MDF) to fuse spectral and image data.

### 2.4. Quantitative Prediction Model Establishment

#### 2.4.1. Division of the Dataset

Before building the model, the 340 datasets were randomly divided in a ratio of 4:1 to obtain 272 samples in the training set and 68 samples in the prediction set.

#### 2.4.2. Elman Neural Network

The Elman neural network has more substantial computing power than the feedforward neural network and adds the context layer to the basic structure of the BP neural network [33]. Its principle is as follows:

(1) $$\{\begin{array}{c} y(i)=g(w_{3}x\left(i\right))\;\;\;\;\;\;\;\\ x\left(i\right){=\alpha [(w}_{1}x_{c}\left(i\right)+w_{2}\left(T\left(i-1\right)\right)]\\ \;\;\;\;\;x_{c}\left(i\right)=x(i-1) \end{array}$$

where i is the iteration time, T denotes the input’s layer input, x is the hidden layer output, and y(i) is the output’s layer output. x_c_ is the context layer output, w_1_ is connection weights from the context layer to the hidden layer, w_2_ is connection weights from the input layer to the hidden layer, w_3_ is connection weights from the hidden layer to the output layer, α is the hidden layer neuron transfer function, and g is output neuron transfer function.

Although the Elman neural networks can solve various linear and nonlinear modeling problems, their initialization weights and thresholds are arbitrary [27]. Hence, this paper invokes the whale optimization algorithm applied to the optimization process of the weight thresholds.

#### 2.4.3. Elman Neural Network Using the Whale Optimization Algorithm

##### Whale Optimization Algorithm

The WOA is a nature-inspired metaheuristic optimization algorithm proposed by Mirjalili in 2016 [29], comprising three main stages in the whale feeding process: encircling prey, bubble-net attack, and searching for prey.

(1) Encircling prey

**Figure 4:**
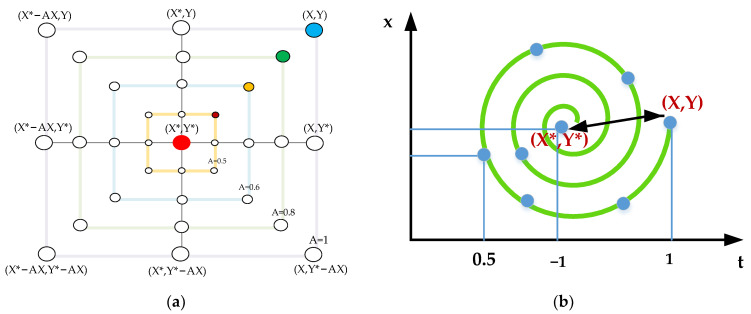
Bubble-net search mechanism implemented in WOA (X* is the best solution obtained so far). (**a**) The shrinking encircling mechanism, and (**b**) spiral updating position.

The Whales need to determine the prey’s location before starting a search as shown in Figure 4a. Assuming that the current position is the optimal solution (target prey), all whales surround it, with the formula updating the prey’s location presented in Equation (2). The following equation represents this behavior:

(2) $$D=|C\cdot X_{p}\left(t\right)-X(t)|$$

(3) $$X(t+1)=X_{p}(t)-A\cdot D$$

where D is the distance between the current whale individual and the current optimal solution, t is the current iteration, X_p_(t) is the position vector of the currently obtained optimal solution, and X(t) is the position vector. A and C are coefficient vectors, calculated as follows:

(4) $$A=2ar_{1}-a$$

(5) $$C=2r_{2}$$

where r_1_ and r_2_ are random vectors in [0, 1], and a is the control parameter.

(2) Bubble-net attacking method

In the bubble-net attack stage, the individual first calculates the distance between itself and the optimal individual in the current population [34]. Then, the humpback whale spits out bubbles while swimming in a spiral trajectory as shown in Figure 4b.

(6) $$D^{\prime }=|X_{p}\left(t)-X(t)\right|$$

(7) $$X(t+1)=D^{\prime }\cdot e^{\mathrm{bl}}\cdot \cos(2\pi l)+X_{p}\left(t\right)$$

where b is the constant that defines the shape of the logarithmic solenoid and l is a random number uniformly distributed in the range [−1, 1].

Since there are two predatory behaviors during the approach to the prey, the WOA selects either encircling the prey or the bubble-net attacking method according to the probability p. The position update equation is as follows:

(8) $$X\left(t+1\right)=\{\begin{array}{cc} X_{p}(t)-A\cdot D & p<0.5\\ D^{\prime }\cdot e^{\mathrm{bl}}\cdot \cos(2\pi l)+X_{p}(t) & p\geq 0.5 \end{array}$$

(3) Search for prey

**Figure 5:**
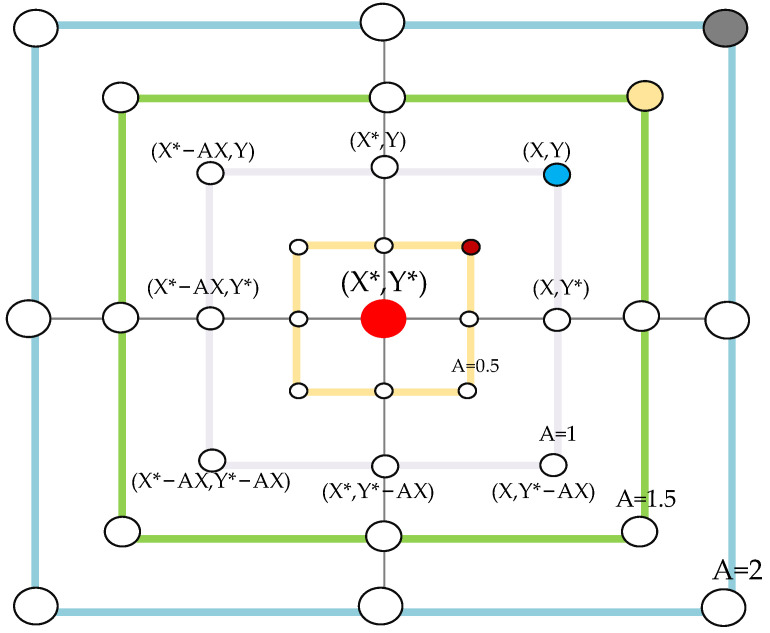
Exploration mechanism implemented in the WOA ( X* is a randomly chosen search agent).

In the initial stage, |A| ≥ 1 to improve the global search ability and avoid moving into local minima. The whale’s location is randomly selected to update the prey’s location as shown in Figure 5, expressed as follows:

(9) $$D^{\prime \prime }=|C\cdot X_{\mathrm{rand}}\left(t)-X(t)\right|$$

(10) $$X(t+1)=X_{\mathrm{rand}}\left(t\right)-A\cdot D^{\prime \prime }$$

where t is the current position of the random individual.

##### WOA-Elman Neural Network

**Figure 6:**
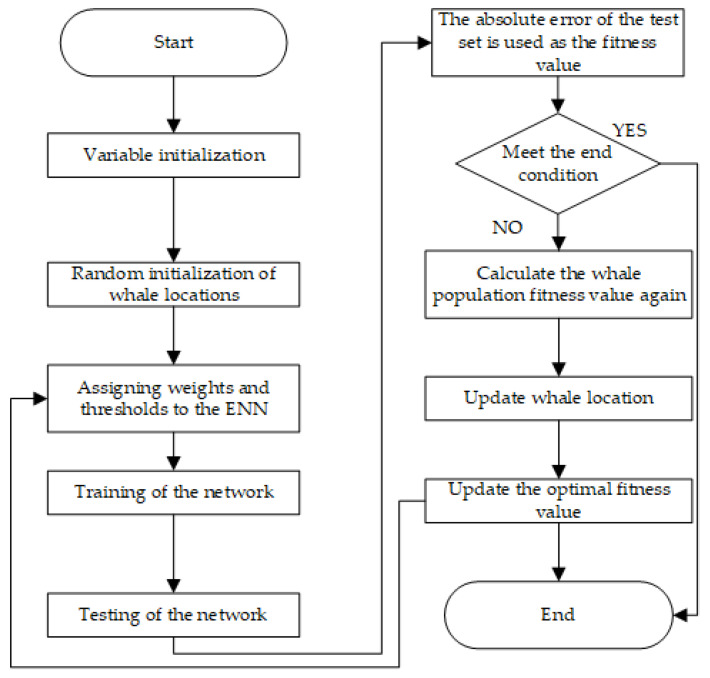
The operation flow of the WOA-ENN model.

The WOA-Elman neural network has three parts: Elman neural network topology, whale optimization algorithm, and Elman neural network prediction. The whale algorithm continuously optimizes the network’s initial weights and thresholds, further improving the global optimization ability of the Elman neural network and preventing the Elman neural network from falling into a locally optimal solution. The WOA-Elman neural network flowchart is illustrated in Figure 6.

#### 2.4.4. Other Quantitative Prediction Models

We compare the advantages of the WOA-ENN model, partial least squares regression (PLSR), and support vector regression (SVR) to establish a quantitative prediction model of moisture content during the green tea fixation process. The PLSR model establishes a regression model by extracting the principal components of the variation information in the independent and dependent variable data [35]. The SVR model is developed utilizing support vector machines (SVMs) [36], using kernel functions to map nonlinear problems in low-dimensional space to linear problems in high-dimensional space, simplifying the problem examined [37].

#### 2.4.5. Model Evaluation

This study uses as model performance indicators the correlation coefficient of the prediction set (Rp), root mean square error of prediction (RMSEP), and relative standard deviation (RPD). When the value of Rp is in the 0~1 range, the smaller the RMSEP, the better the prediction performance. When the RPD value exceeds 1.4, it indicates that the model can be applied. When the RPD is between 1.8 and 2, the model has a good prediction, while when RPD > 2, the model has an excellent prediction ability [13,38,39].

### 2.5. Software

All data processing in this study was performed in Matlab R2021b, and all graphics processed in Visio 2016 and Matlab R2021b.

## 3. Results and Analysis

### 3.1. Optimization of Preprocessing Methods

**Figure 7:**
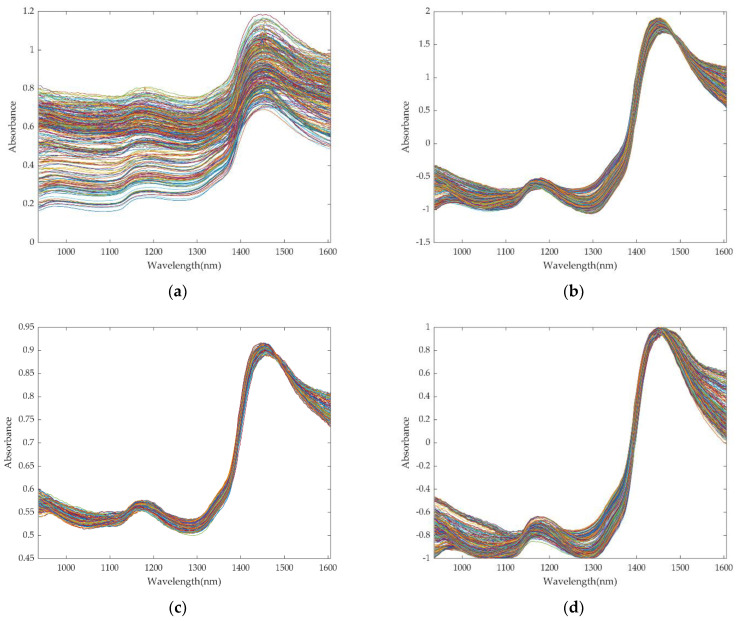
NIR and various preprocessed spectra: (**a**) raw NIR, (**b**) NIR using SNV, (**c**) NIR using MSC, and (**d**) NIR using max–min.

This study retained the spectral data in the 980–1670 nm range due to noise interference beyond the starting and ending of the considered wavelength range. Figure 4a illustrates the raw spectra at 980–1670 nm and reveals two prominent absorption peaks at 1180 nm and 1440 nm. The absorption peak at 1180 nm may be caused by the C–H stretching vibration of CH_3_ and the first overtone of the O–H stretching [25]. The absorption peak at 1440 nm is due to the influence of the first overtone of the stretching vibration of the O–H group in the water molecule in the near-infrared region [40]. Three different preprocess methods were applied to the raw data to reduce noise in the collected near-infrared raw spectra and minimize the influence of noise on the model. The preprocessed spectra are depicted in Figure 7b–d.

The ENN models were established with the preprocessed data for comparison, and the optimal preprocessing method was determined. The results are reported in Table 1. Based on the original spectral data, the Rp value of the prediction set is 0.8664, the RMSEP value is 0.0823, and the RPD value is 1.8897. The RPD value is less than 2, indicating that the model’s robustness is poor due to the surface scattering and the spectral error caused by instrument noise. Our model’s performance was improved after employing the three preprocessing types. Among them, the ENN model established after SNV pretreatment has the best prediction effect, the Rp of the prediction set is 0.9843, and the RPD value is 5.3044, revealing that SNV can effectively improve the model’s accuracy, and reduce the surface scattering and noise. Therefore, SNV was used as the final preprocessing method for subsequent analysis.

### 3.2. Comparison between Single-Sensor Model and Data Fusion Model

After deriving SNV as the optimal preprocessing method (Section 3.1), this section adopts LDF and MDF to fuse the spectral information after the SNV preprocessing and image information. The LDF connects the data obtained by two sensors into a new matrix. The specific operation of MDF is as follows: the image information and the spectrum after SNV were processed by PCA, respectively, then the two sets of data were processed using min–max to eliminate the influence of data size, and, finally, the two data matrices were fused to form a new matrix for the input side of the subsequent prediction model.

**Figure 8:**
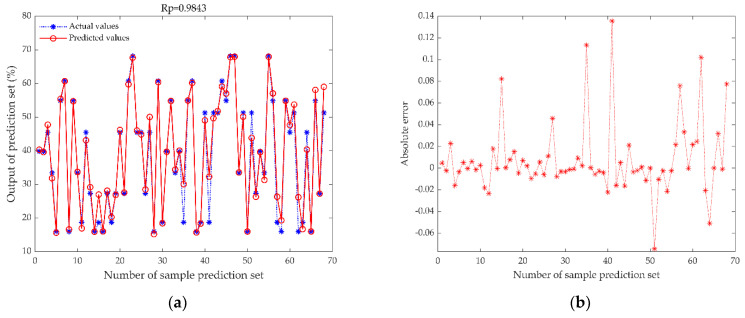

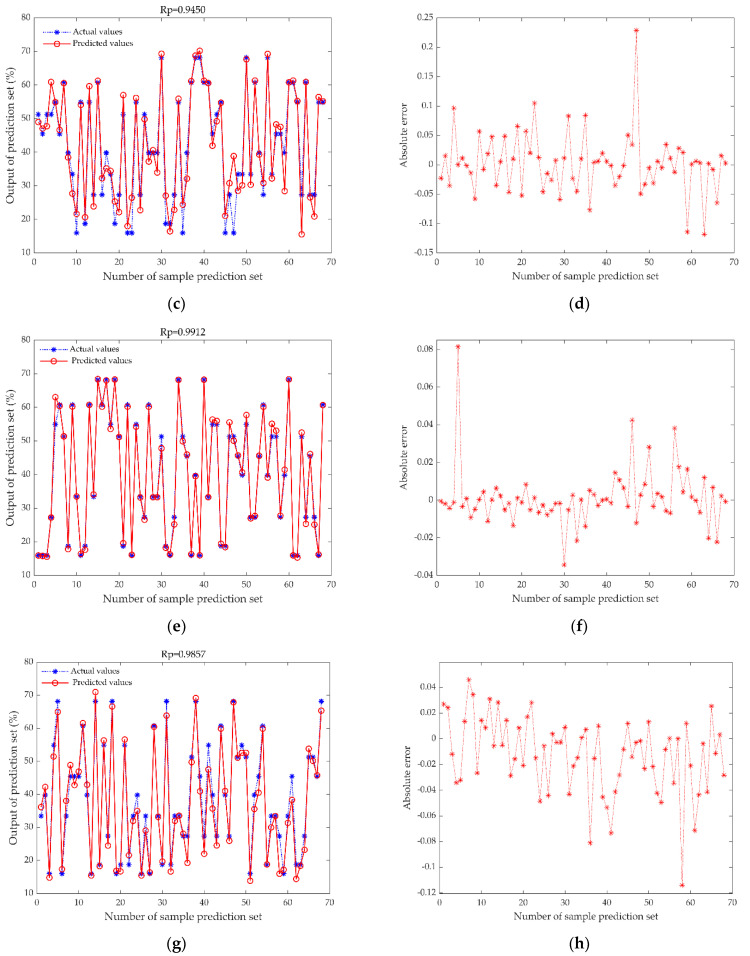
The prediction model of moisture content based on data fusion and single data. (**a**) SNV-ENN model’s scatter plot, (**b**) SNV-ENN model’s absolute error plot, (**c**) CV-ENN model’s scatter plot, (**d**) CV-ENN model’s absolute error plot, (**e**) MDF-ENN model’s scatter plot, (**f**) MDF-ENN model’s absolute error plot, (**g**) LDF-ENN model’s scatter plot, (**h**) LDF-ENN model’s absolute error plot. The actual values are the moisture content measured by the rapid moisture analyzer, the predicted values are the moisture content predicted by different models, and the absolute errors are equal to the predicted value minus the actual value.

The performance of the single-sensor model (CV-ENN and SNV-ENN models) and the data fusion model (LDF-ENN and MDF-ENN models) are presented in Table 2. The SNV-ENN model is the ENN using SNV data to predict moisture content. Figure 8a shows the scattering distributions of the predicted and actual values of moisture content for the SNV-ENN model. Figure 8b is the absolute error plot of the SNV-ENN. The SNV-ENN model’s Rp, RMSEP, and RPD values were 0.9843, 0.0319, and 5.3044, respectively. The CV-ENN model is the ENN using image data to predict moisture content. Figure 8c shows the scattering distributions of the predicted and actual values of moisture content for the CV-ENN model. Figure 8d is the absolute error plot of the CV-ENN. The CV-ENN model’s Rp, RMSEP, and RPD values were 0.9450, 0.0586, and 2.9978, respectively. It can be seen that the model built from NIR spectral data with single-sensor data had a better prediction accuracy than the computer vision-based model. This is because the NIR spectroscopy has a relatively direct response to O–H groups in moisture. In contrast, computer vision can only indirectly characterize the moisture in fixation leaves through color and texture features.

The LDF-ENN model is the ENN using LDF data to predict moisture content. Figure 8g shows the scattering distributions of the predicted and actual values of moisture content for the LDF-ENN model. Figure 8h is the absolute error plot of the LDF-ENN. The LDF-ENN model’s Rp, RMSEP, and RPD values were 0.9857, 0.0313, and 5.4247, respectively. The LDF-ENN model developed using low-level data fusion performs well in terms of prediction. However, such prediction accuracy is not acceptable compared to the single-sensor model (SNV-ENN model), and the performance is not significantly improved, probably because the low-level data fusion contains too much irrelevant information. The MDF-ENN model is the ENN using MDF data to predict moisture content. Figure 8e shows the scattering distributions of the predicted and actual values of moisture content for the MDF-ENN model. Figure 8f is the absolute error plot of the SNV-ENN. The MDF-ENN model’s Rp, RMSEP, and RPD values were 0.9912, 0.0230, and 7.5793, respectively. Compared with the ENN model established by LDF, the ENN model based on MDF obtains better prediction accuracy, indicating that relevant feature information is retained and a lot of useless information is removed in MDF.

### 3.3. Comparison of Elman Neural Network before and after Optimization

In Section 3.2, we compared the effect of the data type on the model’s prediction performance and concluded that the MDF-ENN model had the optimal prediction performance. This section establishes the whale algorithm optimized Elman neural network model under middle-level data fusion (MDF-WOA-ENN). As reported in Table 3, the Rp value of the MDF-WOA-ENN model was 0.9984, and the RMSEP was 0.0090, indicating that the model has a good prediction accuracy. The RPD was 17.9294, suggesting that the model is highly applicable. Compared with the MDF-ENN model, the prediction performance of the MDF-WOA-ENN model was significantly improved. This demonstrated that the whale optimization algorithm effectively enabled the Elman neural network to perform an initial optimization search so that the network obtained the optimal global solution before training. This prevented the network from falling into a local optimum dilemma and improved the network’s prediction performance.

### 3.4. Comparison of MDF-WOA-ENN Model with Mainstream Models

After we constructed the MDF-WOA-ENN model in Section 3.3, it was compared with the mainstream models in tea analysis to prove its excellent performance. The prediction performance of the MDF-PLSR and MDF-SVR models are reported in Table 3. The MDF-PLSR model is the PLSR using MDF data to predict moisture content. Its Rp, RMSEP, and RPD were 0.9517, 0.0488, and 2.9951, respectively. The MDF-SVR model is the SVR using MDF data to predict moisture content. Its Rp, RMSEP, and RPD were 0.9798, 0.0361, and 4.7736, respectively. Compared with the MDF-PLSR, the MDF-SVR model significantly improved performance in all aspects. However, the MDF-SVR model continued to perform poorly, even falling short of the single-sensor-based ENN model, indicating that the choice of prediction model may be more useful than data fusion for quantitative prediction of moisture content in fixation leaves. Compared with mainstream models, the MDF-WOA-ENN model still had the best performance in terms of model prediction accuracy and practical applicability. It proves that the model we built is meaningful.

## 4. Discussion

Moisture content continues to decrease during the fixation process. Moisture content is one of the most important evaluation index in the fixation; besides human experience, the oven drying method is officially measured [41]. However, these methods are either inaccurate or inefficient, and do not achieve fast and accurate measurements. Suitable equipment and models can rapidly and accurately measure the moisture content of fixation leaves to evaluate the fixation degree, which not only ensures the stability of green tea quality but also meets the needs of mass production. 

Previous studies used aroma [6] and color [30] as factors to evaluate fixation; however, the former is susceptible to the environment and the latter lacks comprehensive information. Moisture content is simple to measure and has a positive correlation with green tea quality [42]. As a result, it is possible to measure moisture quickly and accurately to evaluate the degree of fixation. In this study, compared to the NIR spectroscopy model, the machine vision model was worse at predicting moisture. This result may be because the NIR spectroscopy correlates well with the O–H groups in the moisture and images can only capture the color-related component information [19], but the images combined with spectra can add more information about the fixation leaves to better predict the moisture content. The low-level data fusion directly fuses spectral and image data which contain useless information. However, middle-level data fusion removes most of the useless information of the fixation leaves to significantly improve the model’s efficiency and performance. This result is consistent with previous studies [21,43].

**Figure 9:**
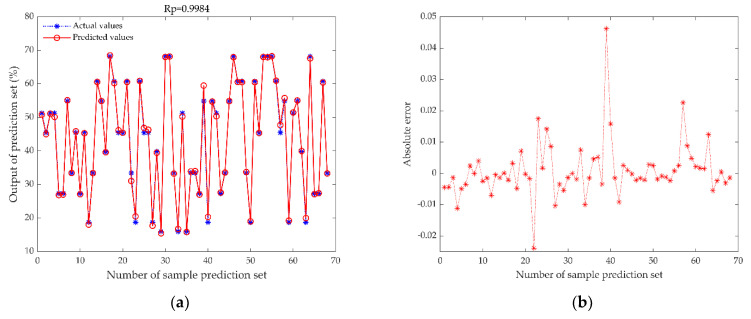

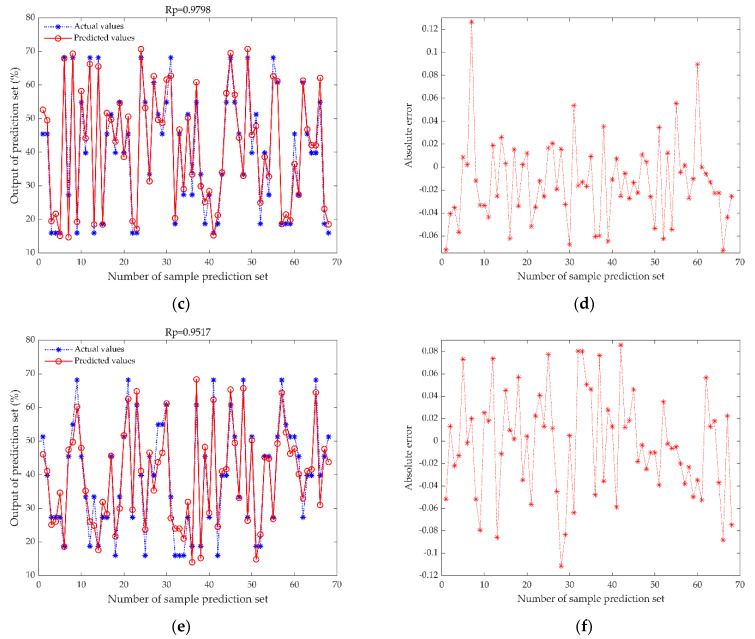
Results of the DF-WOA-ENN model and the competitor models. (**a**) MDF-WOA-ENN model’s scatter plot, (**b**) MDF-WOA-ENN model’s absolute error plot, (**c**) MDF-SVR model’s scatter plot, (**d**) MDF-SVR model’s absolute error plot, (**e**) MDF-PLSR model’s scatter plot, (**f**) MDF-PLSR model’s absolute error plot. The actual values are the moisture content measured by the rapid moisture analyzer, the predicted values are the moisture content predicted by different models, and the absolute errors are equal to the predicted value minus the actual value.

During the model prediction process, ENN prediction results were sometimes unstable, and this is not acceptable. Therefore, we applied the WOA to enhance its stability. Figure 9a,c,e show scatter plots of the measured and predicted moisture content values of the prediction set; the blue asterisks represent the actual moisture values, and the red circles are the predicted moisture values of the three models. Figure 9a shows that the actual measured values and the predicted values of the MDF-WOA-ENN model are the closest, indicating that the model has the best prediction performance. Compared to mainstream models, the MDF-WOA-ENN model uses the whale algorithm to determine the optimal parameters to guide the ENN model for optimal prediction, which has a strong dynamic information processing capability and improves the anti-interference capability and training efficiency.

The fixation is a craft that requires experience and systematic training. However, young people today are unwilling to take the time and patience to learn, and these skills are being lost [30]. Therefore, it is necessary to realize the automation of fixation. To the best of our knowledge, there are few studies on the rapid detection of green tea fixation, and the equipment is complex and time-consuming. Hence, we first use the miniature NIRS to quantitatively predict the moisture content of the fixation leaves in the green tea process. The device is portable, easy to operate, and can be connected to a smartphone via Bluetooth. In the future, if a smartphone operating system can be developed, our model can then be applied to adjust the model parameters online to adapt to different varieties and conditions in green tea fixation.

## 5. Conclusions

This study developed four main models for moisture content prediction of fixation leaves using data fusion based on miniaturized NIR spectral and image data. The WOA algorithm optimized the ENN model’s weight and threshold-finding process. Due to its small size and low environmental requirements, the miniature near-infrared spectrometer in this study was expected to adapt to the complex fixation environment and quickly and accurately predict the moisture content of fixation leaves. From this study, we conclude that:

(1) For the first time, a miniature near-infrared spectrometer was used to collect the spectral data of fixation leaves, and its applicability was verified.
(2) SNV was the optimal spectral preprocessing method, and middle-level data fusion significantly improved the model prediction performance compared to single data.
(3) Compared with classic models such as PLSR and SVR, the ENN model better predicted the moisture content of fixation leaves.
(4) WOA effectively prevented the ENN model from falling into a local optimum and dramatically improved the generalization and robustness of the ENN model. This model attained Rp = 0.9984, RMSEP = 0.0090, and RPD = 17.9294.

## Figures and Tables

**Table 1:** Result of moisture content prediction model for different preprocessing methods.

Preprocessing Methods	Rp	RMSEP	RPD
RAW	0.8644	0.0823	1.8897
SNV	0.9843	0.0319	5.3044
MSC	0.9425	0.0586	2.9978
Max–min	0.9003	0.0391	2.0288

When the Rp value is larger in the range of 0 to 1, and the RMSEP is smaller and the RPD value is larger, the prediction performance is better.

**Table 2:** Model performance indicators based on data fusion and single data.

Types of Models	Rp	RMSEP	RPD
SNV-ENN	0.9843	0.0319	5.3044
CV-ENN	0.9450	0.0586	2.9978
MDF-ENN	0.9912	0.0230	7.5793
LDF-ENN	0.9857	0.0313	5.4247

When the Rp value is larger in the range of 0 to 1, and the RMSEP is smaller and the RPD value is larger, the prediction performance is better.

**Table 3:** Model performance indicators based on middle-level data fusion and single data.

Types of Models	Rp	RMSEP	RPD
MDF-PLSR	0.9517	0.0488	2.9951
MDF-SVR	0.9798	0.0361	4.7736
MDF-WOA-ENN	0.9984	0.0090	17.9294

When the Rp value is larger in the range of 0 to 1, and the RMSEP is smaller and the RPD value is larger, the prediction performance is better.

## Data Availability

The data presented in this study are available on request from the corresponding author.
